# Emotional vernacular boundaries: participation in school culture boosts the emotional logic of rural teachers' rootedness in the countryside—a qualitative study from China

**DOI:** 10.3389/fpsyg.2026.1755357

**Published:** 2026-02-13

**Authors:** Zhen Shuai, Ying Zhang, Jiayin Li, Yunhan Zhou, Chenpeng Xue, Jie Xu

**Affiliations:** Chinese Education Modernization Research Institute, Hangzhou Normal University, Hangzhou, China

**Keywords:** emotional intelligence, qualitative research, rural teacher retention, rural teachers' emotional labor, school culture construction

## Abstract

Paying attention to the emotional labor of rural teachers is a necessary step to enhance their professional happiness and promote the development of high-quality rural education. Based on the theory of emotional intelligence (EI), this study conducted a qualitative research on 13 frontline rural teachers. From the three dimensions of emotional mapping, emotional investment, and emotional resonance, it explored the emotional cornerstone, emotional motivation, and emotional destination of rural teachers in participating in school cultural construction. The study also attempted to explore how to help rural teachers establish a sense of belonging and emotional attachment through emotional labor, ultimately taking root in rural education. The research findings indicate that rural teachers should be assisted in clarifying the direction of their emotional development amid confusion and constructing an emotional map; guided to explore emotional coexistence spaces amid differences and experience emotional investment in the school; and encouraged to cultivate emotional stability through refinement, achieve emotional resonance with the school, and thereby become excellent rural teachers who can take root, actively contribute, and pursue excellence.

## 1 Introduction

Teaching has long been recognized as a profession infused with emotion. As Goodson et al. (2006) notes, teaching is not merely a technical activity but an emotional practice involving deep personal investment. Emotions permeate every dimension of educational life, from interactions with students and colleagues to the teacher's evolving sense of identity and belonging (Schutz et al., 2009). In recent years, growing scholarly attention has been paid to teacher emotions, particularly their role in shaping professional wellbeing, motivation, and classroom engagement (Day and Gu, 2009). Teachers' positive emotions are known to foster resilience and creativity, enabling them to respond more constructively to challenges and maintain effective instructional practices (Chen, 2000). Conversely, chronic exposure to negative emotions—such as frustration, helplessness, or isolation—may lead to burnout, attrition, or emotional disengagement from the profession (Burić et al., 2019; Aydin et al., 2013).

While the emotional lives of urban or elite teachers have been increasingly explored in Western contexts, much less is known about rural teachers' emotional experiences, particularly in non-Western, developing regions such as China. In remote rural schools, teachers often work in emotionally demanding environments shaped by limited resources, administrative burdens, and complex relationships with students and communities. Yet despite these challenges, many rural teachers continue to dedicate themselves to their schools for years, even decades. What drives this commitment? How do teachers form emotional connections with their institutions? What emotional mechanisms underpin their decision to remain, build, and thrive in rural schools?

Recent studies suggest that emotional intelligence (EI)—the capacity to perceive, understand, and manage emotions—is an essential component of teachers' professional growth and retention, especially in challenging environments (Brackett and Mayer, 2003; Taxer and Gross, 2018). However, emotional intelligence is often treated as an individual trait, overlooking the dynamic and contextual nature of emotions as socially constructed within school cultures. In particular, little is known about how emotional labor and emotional belonging emerge through rural teachers' active participation in shaping school culture.

To address this gap, this qualitative study investigates the emotional logic of rural teachers' “rootedness” by examining how they engage emotionally with school culture construction. Drawing on Mayer and Salovey's model of emotional intelligence, this study explores how rural teachers construct emotional maps, develop emotional commitment, and ultimately experience emotional resonance with their schools. Through in-depth interviews with 13 rural teachers across different regions of China, the study aims to illuminate how emotional labor not only sustains teacher retention but also fosters cultural attachment and professional excellence in the rural context.

## 2 Literature review

### 2.1 Rural teacher retention

Rural teacher retention has become a pressing and multidimensional challenge in both global and national education systems (Opoku et al., 2019), especially in remote areas where geographical isolation, limited resources, and shifting demographics intersect. While earlier efforts primarily emphasized financial incentives and staffing policies, contemporary research highlights the dynamic interplay of emotional, organizational, and contextual influences shaping rural teachers' decisions to remain in the profession (Sun and Huang, 2024).

In the macro context of rural education development in China, the issue of teacher retention exhibits a unique trajectory of policy evolution and socio-economic patterns. Teacher retention in rural China is not only influenced by individual motivations and school organizational factors, but also embedded in the macro tensions of urban-rural development imbalance, population mobility, and rural social change. Teachers often make strategic choices between “staying in the hometown” and “moving to the city”. Therefore, placing the Chinese experience in the global research spectrum of rural teacher retention can not only enrich the understanding of multi-factor interaction models in non-Western contexts, but also provide policy references and practical insights for addressing common dilemmas in rural education in countries at different stages of development.

At the early stage of research development, scholars predominantly focused on “structural disadvantages”, highlighting the real-life challenges faced by rural teachers, such as low salaries, poor living conditions, and limited career advancement opportunities (Hammer et al., 2005). As the field progressed, researchers since 2010 have adopted an “incentive-based” perspective, emphasizing teachers' intrinsic motivation and professional identity, while increasingly recognizing the roles of school climate, community relationships, and leadership style (Whaland, 2020). Recent studies have advanced a “multi-factor interaction” model, arguing that teacher retention is shaped by a combination of factors including organizational support, leadership behavior, emotional experience, and opportunities for professional development (Robertson et al., 2020; Korenoski et al., 2021).

In terms of research themes, existing studies mainly focus on three aspects. First is the construction of theoretical frameworks. Many researchers adopt Herzberg's Two-Factor Theory to differentiate between “hygiene factors” (e.g., salary, housing, transportation) and “motivational factors” (e.g., sense of achievement, recognition, and development opportunities). This framework proves effective in explaining both early attrition and long-term retention motivations (Boone, 2018). Second is the identification of key influencing factors affecting teacher retention. Studies consistently find that teachers' achievement goals, experiences of burnout, and contextual factors such as time pressure and student discipline problems are closely linked to their intention to stay or leave (Li et al., 2019). Job satisfaction, perceived visibility of career advancement pathways, and emotional resonance between the individual and school culture are all positively correlated with retention willingness. Third is the exploration of practical interventions and strategies. A range of programs—such as induction initiatives for novice teachers, mentoring systems, emotional support mechanisms, and participatory school culture practices—have been implemented across several countries and shown positive effects in rural contexts.

In terms of methodological approaches, existing research generally falls into three categories: quantitative, qualitative, and mixed-methods studies. Quantitative studies, primarily using surveys, are well-suited for identifying broad patterns in teacher retention (Waterman, 1990). Their advantages lie in large sample sizes, abundant data, and high generalizability; however, they have limited explanatory power regarding complex psychological processes. Qualitative research, conducted through interviews, observations, and case studies, focuses more on teachers' personal narratives and emotional experiences (Patton, 2005). It provides in-depth insights into how teachers form relational ties with their schools and communities, particularly regarding emotional shifts during their rooting process (Whaland, 2020). In recent years, mixed-methods research has gained traction by combining the strengths of both approaches. It bridges the gap between macro-level patterns and micro-level mechanisms, allowing for a more comprehensive understanding of teacher retention.

In the context of the coordinated advancement of rural revitalization and educational modernization, the professional development and emotional well-being of rural teachers have emerged as pivotal issues in enhancing educational quality. Currently, while China's teacher professional development system has bolstered resource provision and skill training through policies like the “Special Post Plan”, it often overlooks the profound influence of teachers' emotional experiences and cultural identity on their professional rootedness. Faced with the dual demands of curriculum reform and the integration of local culture, rural teachers must not only master teaching skills but also adapt and integrate emotionally into the rural educational ecosystem. Therefore, focusing on teachers' emotional labor and exploring the intrinsic connection between their emotional development and professional commitment has become a crucial research direction to address the shortcomings of the current teacher support system and achieve sustainable development in rural education.

Despite notable progress, several research gaps remain in the field of rural teacher retention. First, existing studies are predominantly based on English-speaking contexts, with limited empirical research focused on China—particularly in remote ethnic regions and areas of extreme poverty. Second, most studies adopt cross-sectional designs, lacking longitudinal tracking of emotional, identity, and behavioral changes across the teacher's professional life cycle. Third, some research overlooks the agency of teachers as central actors in education, placing excessive emphasis on institutional-level “external empowerment” without adequately exploring how teachers actively construct emotional bonds with their schools and communities. Finally, theoretical frameworks regarding how teachers achieve emotional belonging and professional identity through participation in school culture remain underdeveloped, lacking robust theoretical models and empirical validation. At the theoretical level, there is an excessive reliance on Western classic theories and a lack of localized and integrated theoretical frameworks rooted in China's specific local social, cultural, and institutional backgrounds. Existing models are mostly static analyses and fail to depict the dynamic evolution of needs and motivations in the teaching profession. In terms of influencing factors, although multi-level elements have been identified, the complex interaction mechanisms, causal paths, and specific connections between macro and micro factors still belong to the “black box”. In terms of research methods, there is a serious lack of long-term tracking studies to capture key turning points in retention intentions. Cross sectional design dominates, making it difficult to reveal the dynamic decision-making process. The application of mixed methods is often superficial and lacks true integration and mutual interpretation of quantitative breadth and qualitative depth.

### 2.2 Teacher retention from an emotional intelligence perspective

Research on emotional intelligence (EI) can be traced back to the early 20th century, when scholars began to emphasize “non-cognitive factors” (Sharma, 2008). In 1920, Thorndike introduced the concept of social intelligence, which emphasized individuals' ability to understand and manage interpersonal relationships (Kosmitzki and John, 1993). Later, psychologists such as Wechsler and Leeper contributed to this foundation by proposing the importance of non-intellective factors and emotional thinking, laying the groundwork for the emergence of the EI construct (Wechsler, 1943; Leeper, 1948). In 1990, American psychologists Salovey and Mayer formally defined emotional intelligence as a part of social intelligence, involving the ability to perceive, assess, and express emotions, as well as to regulate them in ways that enhance thinking and behavior (Salovey and Mayer, 1990). In 1995, Daniel Goleman popularized the concept through his best-selling book Emotional Intelligence, proposing a five-component model—self-awareness, self-regulation, motivation, empathy, and social skills—that brought the concept into the mainstream and sparked widespread interest (Goleman, 1995, 2001). Around the same time, Bar-On developed the concept of emotional quotient (EQ) and introduced the EQ-i assessment, conceptualizing EI as a set of personality traits including adaptability, stress management, and mood. This trait model stood in contrast to Salovey and Mayer's ability model, which emphasized cognitive processing of emotional information (Bar-On, 1997). Since then, emotional intelligence theory has evolved into three major models: the ability model (Salovey and Mayer), the mixed model (Goleman), and the trait model (Bar-On).

In the study of teacher retention, cultural belonging and emotional logic are closely intertwined and mutually causal, together constituting the core mechanism that influences teachers' decisions to stay or leave. Cultural belonging provides a meaningful framework and social soil for teachers' emotional investment and rooting. A strong sense of regional cultural identity can significantly enhance teachers' emotional security and professional meaning, thereby translating into a more stable willingness to stay. At the same time, teachers experience and integrate into local culture through daily emotional practices. Positive emotional experiences accelerate their cultural identity and identity transformation, forming an interactive cycle between emotion and culture. This theoretical perspective reveals that teachers' long-term commitment not only depends on external guarantees but also on their dynamic process of actively constructing meaning and achieving self-identity through emotional bonds in a specific cultural field. Ignoring the interactive relationship between the two makes it difficult to fully explain the vastly different career choices made by teachers under similar structural conditions. In the present study, considering the sociocultural particularities of rural China, we adopt the refined hierarchical model of emotional intelligence developed by Mayer et al. (2000), which delineates four progressive levels: emotional perception and expression, emotional facilitation of thinking, emotional understanding and analysis, and emotional regulation and management (Mayer et al., 2000).

The teaching profession demands a high level of emotional regulation, making emotional intelligence (EI) a crucial theoretical tool for understanding teacher behavior. Scholars have widely drawn on the ability model of EI proposed by Salovey and Mayer (1990), which conceptualizes EI as a subsystem of social intelligence involving the ability to perceive, understand, and regulate one's own and others' emotions, and to apply this information effectively in thinking and behavior. Within the field of teacher emotion research, Brackett and Mayer (2003) emphasized that teachers' emotional intelligence demonstrates strong validity in predicting self-efficacy, classroom management skills, and the quality of instructional interactions. They identified EI as a key variable in understanding professional performance and emotional labor capacity (Brackett and Mayer, 2003). Palomera et al. (2017) further argued that EI should be considered a fundamental competency in teacher education, as it is closely linked to job satisfaction and stress coping strategies, while also showing a significant negative correlation with teachers' sense of burnout and loss of mission. Other researchers, such as Taxer and Gross (2018), have connected EI with emotional labor, showing that teachers' surface and deep emotional regulation strategies during daily instruction are significantly influenced by their levels of emotional intelligence. These, in turn, affect their professional well-being and the quality of student–teacher interactions (Taxer and Gross, 2018). Empirical studies have shown that teachers with high emotional intelligence are more likely to engage in deep emotional regulation in response to classroom stress, which helps reduce burnout and enhance teaching satisfaction. For example, (Chan 2004, 2006); Aliyev et al. (2013) conducted large-scale survey studies and found that teachers with higher EI are better able to adapt to the dynamic emotional demands of the profession and maintain a positive psychological state under occupational stress (Chan, 2004, 2006).

In summary, current research on rural teacher retention has made notable progress in addressing structural factors and career development pathways. However, significant gaps remain in the emotional and cultural dimensions of this issue. Specifically, first, emotional intelligence is often treated as a static individual trait, overlooking its dynamic construction in relation to school culture and emotional labor within specific educational contexts. Second, existing studies tend to rely on quantitative path models, with limited attention to how teachers construct “emotional maps” through subjective experience, and rarely explore the deeper role of school culture as a mechanism that activates and regulates emotional processes influencing teachers' willingness to stay. Third, when analyzing why rural teachers choose to remain, the literature largely stays within a static “compensation—development” framework, lacking systematic exploration of the dynamic emotional logic chain involving “emotional momentum—cultural attachment—resonant belonging.” In response, this study proposes a novel perspective that conceptualizes emotional intelligence as a relational resource that is continuously constructed within context. By introducing a triadic framework of “emotional mapping—emotional commitment—emotional resonance”, the study aims to illuminate the emotional mechanisms underlying rural teachers' place attachment. Theoretically, this approach extends the applicability of emotional intelligence into culturally embedded educational settings; methodologically, it moves beyond the constraints of traditional quantitative approaches by emphasizing the power of qualitative analysis to capture teachers' subjective emotional experiences. This provides both a robust psychological foundation and a culturally grounded perspective for promoting emotionally anchored teacher retention strategies.

## 3 Conceptual framework

Since the 1980s, emotional intelligence (EI) has attracted significant academic interest, giving rise to a range of theoretical frameworks. Among the most influential are those proposed by American scholars Mayer, J.D. and Salovey, P., who conceptualized EI as a type of ability. In 1990, they synthesized existing literature and put forward the first formally published definition of emotional intelligence, which for the first time summarized emotional intelligence as an independent component of intelligence, and emphasized that emotional intelligence includes the correct evaluation of emotions, the appropriate expression of emotions, and the adaptive regulation of emotions (Neubauer and Freudenthaler, 2005). According to Salovey and Mayer (1990), individuals with high emotional intelligence exhibit greater flexibility in emotion use, enhanced creative thinking, improved attentional control, and a stronger capacity for self-and other-motivation (Salovey and Mayer, 1990).

However, their initial model primarily focused on emotional perception and regulation, with limited attention to how emotions influence cognitive processes. To address this limitation, Mayer and Salovey revised their model in 1997, offering a more structured, four-branch framework of EI: the ability to perceive, assess, and express emotions, the ability of emotion-facilitated thinking, the ability to understand and analyze emotions, and the reflexive regulation of emotions. This framework was further refined in 2000 by Mayer, Salovey, and Caruso, who emphasized the role of EI as a mental ability intimately connected with cognitive functioning. They formalized a hierarchical model comprising the same four progressively complex branches: perception and expression of emotion, emotional facilitation of thinking, emotional understanding, and emotional regulation (Mayer et al., 2000).

In the research framework of rural teacher retention, “participation in school culture” serves as a key independent variable, which ultimately exerts a positive influence on the core dependent variable of “rural belongingness” through the mediating variable of “emotional enhancement”. Specifically, teachers can gain a greater sense of meaning, achievement, and collective support in their work through practices of “participation in school culture” such as participating in decision-making, integrating into collective activities, and jointly building educational philosophies. These positive experiences directly promote teachers' “emotional enhancement”, including the application of emotional intelligence, the accumulation of positive emotions, and the deepening of emotional commitment. This internal “emotional enhancement” not only buffers work pressure and job burnout, but more importantly, it enables teachers' emotional experiences to deeply connect with rural school and community life, gradually internalizing their identification and attachment to the region, thereby forming a solid “rural belongingness”. Therefore, emotional enhancement is a key psychological mechanism connecting external cultural participation behaviors with the formation of internal belongingness. This logical path of “behavior-emotion-identity” indicates that intervention measures aimed at improving teacher retention cannot only focus on the formal level of institutional participation, but also need to pay attention to the stimulation, nurturing, and sublimation of teachers' emotions during the participation process. Building on this model and considering the emotionally charged nature of rural teachers' work, the emotional processes involved in their engagement with school culture construction and rural education can be analyzed through these four interrelated and evolving dimensions.

**Figure 1:**
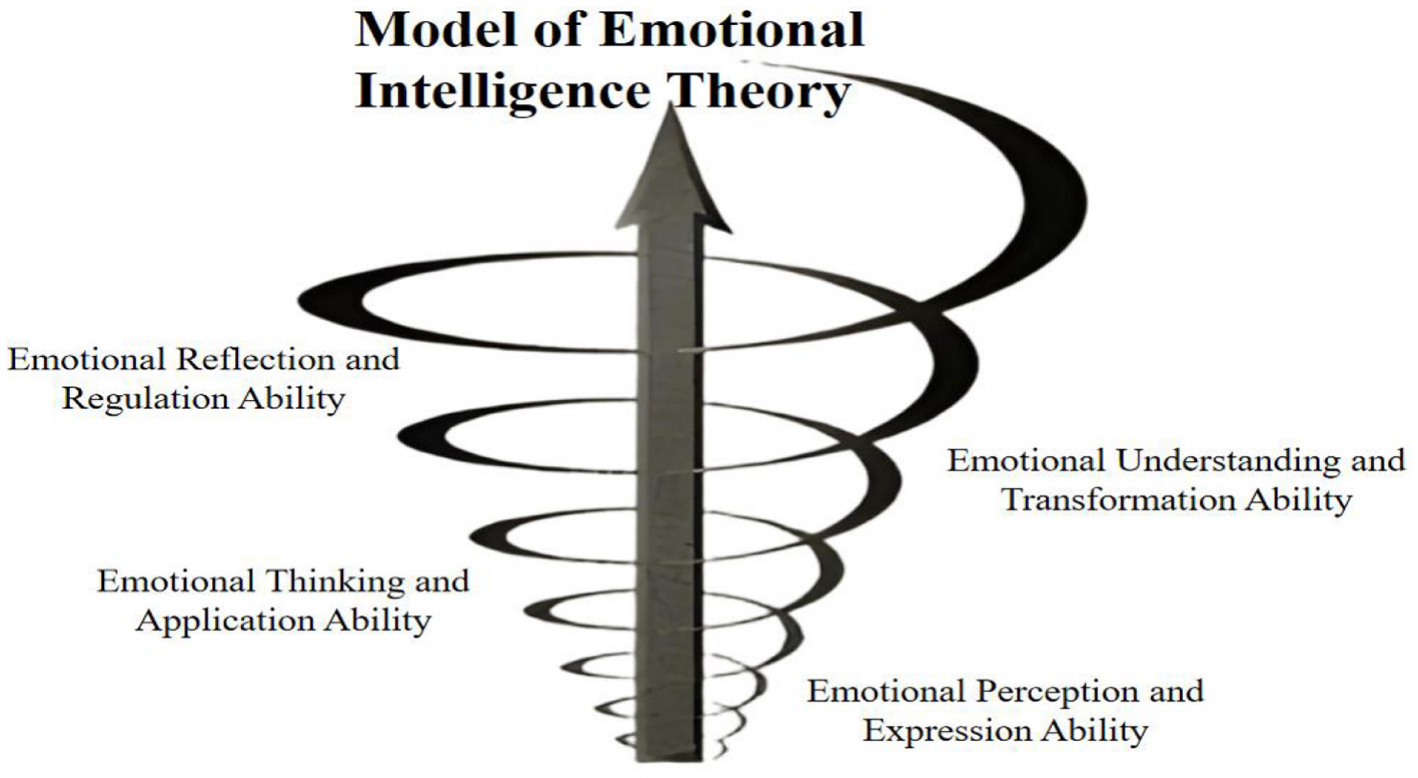
Model of emotional intelligence theory.

We will apply the four-branch model of emotional intelligence as the theoretical framework to analyze the themes summarized from teachers' experiences and reveal the formation process of their emotional roots (see Figure 1).

**Emotional Perception and Expression Ability:** Rural teachers demonstrate a strong capacity for emotional perception during their involvement in school culture construction. They are adept at recognizing both their own emotional states and those of others, enabling them to establish effective communication and support networks. This emotional awareness enhances their active participation in shaping the school culture and reflects their deep-rooted commitment to education in rural contexts.

**Emotional Thinking and Application Ability:** By accurately identifying emotions in themselves and others, rural teachers are able to extract key information and enhance cognitive processing through emotional insight. This allows them to approach challenges in school culture construction more effectively, leveraging emotional intelligence to optimize problem-solving strategies. In doing so, they not only address immediate school-level issues but also contribute to broader solutions in rural education.

**Emotional Understanding and Transformation Ability:** Rural teachers recognize the dynamic and multidimensional nature of emotions that emerge throughout the process of school culture construction. They are capable of discerning the characteristics and implications of various emotional states, and they employ emotional intelligence strategies to transform these emotions constructively—for example, channeling anxiety into motivation or reframing frustration as an opportunity for growth. This emotional adaptability extends to their daily professional lives, enabling them to navigate negative emotions with resilience and a constructive mindset.

**Emotional Reflection and Regulation Ability:** Following their participation in school culture initiatives, rural teachers adopt an open and accepting attitude toward the full range of emotional experiences. Through reflective regulation and metacognitive strategies, they enhance positive emotional states and mitigate negative ones, fostering a sustainable cycle of emotional labor. This process empowers them to continuously infuse their educational practice with emotional energy, thereby strengthening their long-term contribution to rural education.

## 4 Research design and implementation

### 4.1 Research question

This study aims to explore the following core issues: within the cultural context of rural Chinese society, how does the emotional experience of rural teachers undergo a dynamic evolution through their participation in school culture construction? How does this process, mediated by the abilities of perception, application, understanding, and regulation of emotional intelligence, ultimately shape their professional commitment to rural education and sense of regional belonging? Specifically, the research will focus on: the emotional perception map and meaning construction mechanism of teachers in school cultural practice; the specific pathways of emotional ability in resolving professional dilemmas and transforming negative experiences; and how the interaction between emotional identity and local cultural embedding facilitates the deep transformation of teachers from “professional adherence” to “taking root in life”. By answering these questions, this study attempts to reveal the complex emotional sociological logic behind the retention of rural teachers, providing theoretical and practical paths based on emotional development and cultural integration for the revitalization of local education.

### 4.2 Research methods and objects

This study adopts a qualitative research design with an interpretive orientation and employs the triangulation method to enhance the validity and explanatory depth of the research. Through the cross-validation of in-depth interviews, participatory observation, and material analysis, the study systematically collects data on rural teachers' emotional experiences in school culture construction. Interviews focus on teachers' subjective narratives and meaning construction, observations record their emotional expressions and interaction patterns in actual situations, and materials provide evidence of practical contexts. These three data sources complement and contrast with each other in the analysis process, presenting the complexity of emotional phenomena from multiple perspectives and avoiding the limitations of a single method through cross-validation, thus achieving a more comprehensive and credible interpretation of teachers' emotional experiences. Grounded in this qualitative paradigm, the present study adopts school culture construction as the analytical entry point and employs emotional intelligence theory to investigate and interpret the emotional motivations behind rural teachers' decisions to stay in, contribute to, and thrive within rural educational settings. A combination of purposive sampling and snowball sampling was used to recruit exemplary frontline rural educators rooted in the front line. This study explored the emotional experiences and willingness to stay of 13 rural teachers in school culture construction through in-depth interviews. The demographic and professional characteristics of the interviewees are summarized in Table 1. The sample of interviewees was equally divided between males and females; their teaching experience spanned from 2 to 25 years, with seven individuals having more than 10 years of experience; their educational stages covered primary school, junior high school, and high school; their school locations included rural areas in provincial capitals and non-provincial capitals; and their job positions included both frontline teachers and administrative staff. This sample possesses good diversity and representativeness, providing a multi-dimensional and multi-level empirical foundation for understanding the emotional experiences and career choices of rural teachers. Through unstructured, open-ended interviews, the study aims to construct an emotional framework informed by teachers' engagement in school culture, and to analyze how emotional factors can be leveraged to improve rural teacher retention. Recognizing that the emotions arising from teachers' participation in school culture construction are influenced by both subjective and objective factors—including educational background, teaching experience, role type, teaching stage, and professional identity—the study followed the principle of maximum variation sampling to ensure the representativeness and diversity of its findings. The participant selection criteria were as follows: (1) teachers at the elementary, middle, or high school levels who have taught in rural areas for at least 1 year; (2) rural teachers who have participated in, or expressed a willingness to participate in, school culture construction and who possess distinct perspectives or insights on the topic; and (3) rural teachers in both administrative and non-administrative positions.

**Table 1:** Information table of interviewed teachers.

**No**.	**Gender**	**Teaching experience (unit: years)**	**Type of school**	**Position held**
A1	Male	12	Provincial capital city	Subjects team leader
			Rural primary school	
A2	Male	16	Provincial capital city	Logistics director
			Rural primary school	
A3	Female	25	‘Provincial capital city	Vice principal
			Rural elementary school	
B1	Male	5	Provincial capital city	–
			Rural junior high school	
B2	Female	7	Provincial capital city	–
			Rural middle school	
C1	Female	4	Non-capital cities	–
			Rural primary schools	
C2	Male	19	Non-provincial capital cities	Vice principal
			Rural primary school	
C3	Female	14	Non-provincial capital cities	–
			Rural primary schools	
D1	Female	3	Non-provincial capital city	–
			Rural junior high school	
E1	Male	16	Non-capital city	Moral education director
			Rural junior high school	
F1	Female	2	Non-provincial capital city	–
			Rural high school	
F2	Male	16	Non-capital city	Subjects team leader
			Rural high school	
F3	Female	7	Non-provincial capital city	–
			Rural high school	

### 4.3 Data collection

This study employed a semi-structured interview approach to collect qualitative data. Each participant engaged in a 30-min 60-min in-depth interview guided by a predefined interview outline, in order to explore the emergence and evolution of emotions experienced by rural teachers as they participated in the construction of school culture. Specifically, the researcher designed an open-ended interview outline based on the theoretical framework of emotional intelligence, and resorted to the rural teachers' answers to the interview questions in order to intended to elicit authentic emotional experiences in school culture construction. The interviews aimed to uncover how these emotional experiences influence teachers' sense of professional identity and contribute to their sustained commitment to front-line educational work in rural settings.

This interview focuses on the emotional experience and transformation mechanism of rural teachers in the process of participating in school culture construction. The core issues revolve around the four dimensions of emotional intelligence: firstly, exploring teachers' perception and expression abilities of self and others' emotional changes in the process of cultural practice; secondly, analyzing how they promote problem-solving based on emotional cognition, that is, the application ability of emotions in thinking and decision-making; thirdly, examining teachers' abilities to recognize, categorize, and positively transform complex emotions, that is, the ability to understand and integrate emotions; finally, discussing how they adjust and reflect in the tension of multiple emotions to balance self and others, positive and negative emotions. The main thread running through it all is to inquire into how these dynamic emotional experiences ultimately shape and deepen their professional emotions, thereby revealing the deep mediating role of emotional participation in school culture construction and rural teacher retention.

While the interview questions cover the rural teachers' knowledge of school culture construction, their emotional perception in the process of school culture construction, and the transformation of their emotions in the process of school culture construction, the interview questions also pay special attention to the logic of the rural teachers' emotions in the process of participating in the school culture construction to promote their thinking, the reflective regulation of their emotions in the process of school culture construction, and the impact of these emotions on their professional emotions. The impact of these emotions on their professionalism like: What changes in emotions do you perceive in yourself and others (colleagues, students, parents, etc.) in the process of achieving the goals of school culture building? (Emotional Perception and Expression Ability) Based on your perception of your own and others' emotions, how did you grasp the key messages and actively solve the problems you encountered in building school culture and in rural education? (Emotional Thinking and Application Ability) Can you identify the characteristics of various complex emotions generated in the process of participating in the construction of school culture, and categorize and transform them in a positive direction? (Emotional Understanding and Transformation Ability) In the process of building school culture, how do you accept all kinds of emotions and try to strike a balance between self and others, positive and negative emotions? (Emotional Regulation and Reflection Ability) In the above emotional experiences, what are the effects on your professional emotions?

### 4.4 Data analysis

First, we adopted the three-stage coding process of the inductive method, which distilled three core themes: emotional maps, commitment, and resonance. Then, we used the four-branch emotional intelligence model as a theoretical framework to conduct deductive analysis and interpretation of these themes, which are specifically presented in the research results.

More than 15,000 words of textual data can be collated through qualitative interviews with rural teachers, which are imported into NVivo11 software for qualitative analysis such as high-frequency word extraction, open coding, principal axis coding and selective coding, which can form the nodes and key themes about rural teachers' emotions about participating in school culture building. Subsequently, based on the theoretical model of emotional intelligence proposed by Meyer and Salovey, the qualitatively coded data were deductively categorized into the four major domains of perception and expression of emotion, assimilating emotion in thought, understanding and analyzing emotion, reflective regulation of emotion. Therefore, we could analyze and reflect on the emotions of rural teachers' participation in school culture construction, and explore the characteristics of the teachers who have excelled in the construction of school culture. Such research can help search for the emotional logic of rural teachers' participation in education, and to make more rural teachers willing to put down roots in the countryside, and to become excellent teachers in the countryside who are able and willing to “to remain rooted, to actively contribute, and to strive for excellence”. It should be pointed out that, in the process of organizing and analyzing the interview data, in order to ensure the authenticity of the interview data, it is necessary to use the triangulation of mutual evidence, from the perspective of the correlation between the front and back of the interview data of the same interviewee, and other perspectives of the coded information, so as to more comprehensively qualitatively generalize the information obtained in the interview text. So that can ensure the research credibility and persuasiveness, and to reveal the complexity and diversity of the emotions of the rural teachers' participation in the construction of the school culture. The complexity and diversity of the emotions of rural teachers' participation in school culture building.

To enhance the rigor and methodological transparency of qualitative analysis, the following Table 2 visually presents the complete process from open coding to axial coding and selective coding, clearly illustrating the path of core theme extraction.

**Table 2:** Coding process.

**Coding phase**	**Operating steps**	**Core output**	**Encoding logic description**
Open coding	1. Import over 15,000 words of interview text into NVivo11 software; 2. Extract emotion-related original sentences one by one and perform conceptual annotation; 3. Eliminate repetitive and meaningless concepts, and merge semantically similar expressions	Original concepts: 1. Work confusion, task anxiety, and uncertainty about change; 2. A sense of achievement from having one's ideas recognized, and a sense of collective belonging; 3. Transforming negative emotions into motivation, emotional balance strategies—a resonance with the school's progress and decline, and a connection to local emotions	Breaking the original structure of the text, focusing on the core dimension of “emotional experience”, fragmenting and preliminarily categorizing the raw data to ensure objectivity in the coding process and comprehensiveness in data coverage
Axial encoding	1. Based on the theoretical framework of emotional intelligence (perception-application-understanding-regulation), conduct a correlation analysis on the results of open-ended coding; 2. Explore the causal, subordinate, and associative relationships among various concepts, and cluster them to form category clusters	Core categories: 1. Emotion perception and recognition (capturing basic emotions such as confusion, anxiety, and sense of achievement); 2. Emotion application and problem solving (leveraging positive emotions to facilitate the implementation of work); 3. Emotional transformation and reconstruction (transformation of negative emotions into motivation); 4. Emotional regulation and balance (regulation strategies such as reflection, task decomposition, etc.); 5. Organizational emotional support (school trust atmosphere, idea recognition mechanism); 6. Emotional resonance and belonging (deep emotional connection between individuals and schools/villages)	Establish the inherent logical connection between fragmented concepts, integrate scattered ideas into systematic clusters, and build an intermediate analytical bridge connecting “emotional experience—behavioral response— result orientation”
Selective coding	1. Filter core categories and establish “the emotional logic of rural teachers taking root in the countryside” as the core axis for coding; 2. Integrate category clusters and refine core themes that can unify all raw data; 3. Review original interview texts to verify the adaptability of core themes to data, and supplement marginal cases to enrich the connotation of themes	Core themes: 1. Emotional map—the rooted emotional cornerstone (corresponding to the emotional closed loop of “perception-recognition-transformation-regulation”); 2. Emotional commitment—the emotional driving force of dedication (corresponding to the interactive mechanism of “organizational support-belonging and identity”); 3. Emotional resonance—the emotional destination of excellence creation (corresponding to the deep connection state of “individual-school-village”)	Focus on the core research issues, connect all categories and raw data with the core theme, form a complete theoretical explanation framework, and ensure the inclusiveness and representativeness of the theme toward the research phenomena

## 5 Research findings and analysis

To avoid the mechanical alignment of interview results with pre-existing theoretical frameworks—and thereby reduce the risk of merely “verifying theories with facts”—this study adopts an inductive approach in the presentation of findings. Specifically, a three-level coding process was employed to distill the emotional trajectories of rural teachers participating in school culture construction. These trajectories were synthesized into three core thematic dimensions: constructing emotional maps, experiencing emotional commitment, and realizing emotional resonance, etc. The theory of emotional intelligence was used to analyze and interpret them, so as to summarize the emotional boundaries of the rural teachers' rooting in the countryside and analyze how to make use of the teachers' emotional labor to urge more rural teachers to remain rooted, actively contribute, and strive for excellence.

### 5.1 Emotional maps—the emotional cornerstone for rural teachers to remain rooted

Emotional labor represents the underlying logic that drives individual behavior, with variations in emotional experience leading to differentiated actions and, consequently, diverse outcomes. As noted by Alicia A. Grandey, a professor of psychology at Pennsylvania State University, employees who operate within a positive emotional environment tend to exhibit higher job satisfaction, reduced stress levels, and a lower intention to leave their positions (Grandey, 2000). Findings from the interviews suggest that rural teachers' emotional development throughout their participation in school culture construction is, at its core, an ecological process of emotional-cognitive reconstruction and educational emotional remodeling. This dynamic process can be conceptualized as the drawing of “emotional maps”—a metaphorical representation of how teachers navigate, interpret, and reshape their emotional experiences in response to their professional environments and responsibilities.

Specifically, first of all, the enhancement of rural teachers' professionalism in the rural education field begins with their accurate perception and expression of the emotions generated in their work. In practice, in the process of subjective cognition and inter-subjective interaction, rural teachers always decode the emotions they feel. “*When the school leaders started to talk about building the school culture, I felt confused in the face of the challenges of the new task, and I felt anxious because I didn't know what I could do in it.” (C1)*; Secondly, based on the emotional awareness of self and other, rural teachers resorted to the interactive interpretation of emotional cognition and gradually constructed a logical model of emotion generation in the embodied experience of emotional flow, i.e., through metacognitive monitoring, they knew why they produced a certain emotion. “*I had noticed that myself and other teachers had some negative emotions such as anxiety and resistance in the process of building school culture, and I often wondered, “Why do we have such emotions?” Upon reflection, I realized that such emotions usually stemmed from a sense of uncertainty about the change, or concerns about the additional workload.” (E1)*; Again, rural teachers accomplished an evolution of emotions based on a deep understanding of the various emotions that arose from their participation in the school culture process. “*The transformation of negative emotions to positive emotions is a gradual process, for example, when the condensed school system culture did not achieve the expected results, my emotions were in a completely negative state, and I thought that my efforts and those of the teachers were not rewarded. Later I realized that the reason for the negative emotions was that I wanted to make the school have a more grounded and democratic management system, and I gradually began to transform the negative emotions into motivation for future improvement.” (C3)*; Finally, some rural teachers would regularly reflect on and evaluate their emotions generated in the midst of participating in school culture building, and regulate their productivity through their emotions. “*I would regularly and consciously reflect on and assess my emotions generated in the midst of participating in school culture building. Through self-reflection, I am able to treat all kinds of emotions with a more tolerant mindset and recognize the reasons for my emotions and the changes in my emotions, so that I can adjust my emotional state in time to improve my work efficiency. For example, when feeling anxious or stressed, I would adopt the technique of breaking down tasks to reduce stress.” (F3)*.

The emotional development process of rural teachers involves a progression from the perception of basic emotions, to emotional analysis, transformation of negative emotions into positive ones, and ultimately to reflective emotional regulation aimed at improving work efficiency. This process can be conceptualized as the construction of an “emotional map”. Drawing on Mayer's theory of emotional intelligence, rural teachers engage in symbolic interpretation of educational emotions through emotional perception and expression. They embody educational wisdom through emotional reflection and application, establish value-oriented professional identities through emotional understanding and transformation, and construct cognitive frameworks for emotional navigation through emotional regulation and introspection. Once this emotional map is established, rural teachers are better positioned to engage in emotional labor within a psychologically safe zone, fostering an educational sentiment deeply rooted in rural life. Therefore, cultivating the emotional intelligence of rural teachers should be prioritized through active involvement in school development and related educational activities. Such engagement helps them build an emotional network grounded in the context of rural education, enabling them to construct personal emotional maps. In turn, this process supports the formation of committed, resilient, and high-quality rural teachers who remain rooted in the countryside.

### 5.2 Emotional commitment—the emotional momentum for rural teachers to actively contribute

From the perspective of organizational behavior, emotional commitment—encompassing rural teachers' organizational identity, emotional attachment, and professional dedication—constitutes a complex psychological contract that plays a vital role in sustaining their long-term engagement in rural education (Meyer and Allen, 1991). American sociologist Eisenberger highlights that when educators perceive care, support, and recognition from their organization (i.e., the school), they tend to develop a sense of emotional obligation and a motivation to reciprocate through behavioral contributions. This emotional indebtedness can be transformed into the driving force of educational practice through emotional labor (Eisenberger et al., 1986). Indeed, within the context of rural education and under the framework of social exchange and reciprocity (Blau, 1964), teachers who feel valued and respected by their institutions are more likely to develop a strong sense of belonging and identification with rural schools and the broader mission of rural education.

Most of the teachers in the study said that they develop positive emotions when they are recognized from various sources, which makes them more willing to participate in school building. “*Our principal insists that every teacher be involved in the whole process of school culture construction. When the ideas I put forward are adopted and utilized in the final school culture system, a sense of accomplishment and pride will arise in me, and I will feel that I have truly become a part of this school, which makes me firmly believe in dedicating myself to the school.” (A2); “When my ideas are recognized by my colleagues and leaders, I will feel a sense of responsibility and mission inspired, and this emotion drives me to be more active in communicating with my colleagues and to be more deeply involved in all the work of the school.” (F1)* In addition, transferring the positive emotional experiences generated in participating in school culture building to daily work in rural education also stimulates teachers' work efficiency and professional identity. “*By applying the positive emotions gained from participating in school culture building to my educational work, I feel more motivated to design more interesting and meaningful teaching content to motivate my students to learn. When I encounter difficulties, these positive emotions will also make me feel that I am not fighting alone, but that there is a strong collective force behind me, and I will be more willing to maintain a close cooperative relationship with my colleagues in my work to support and encourage each other.” (F2)* Using the theory of emotional intelligence to analyze, rural teachers try to explore the mechanism of various emotions through the ability to think and use emotions (e.g., positive emotions will be stimulated when the results of school culture construction are affirmed by parents, leaders, and students, and vice versa, negative emotions will be developed), can ultimately, use positive emotions to enhance the efficacy of the work of rural education.

In other words, rural teachers' willingness to remain in and contribute to rural education is closely tied to the level of emotional support they receive. Therefore, it is essential to make rural teachers feel a strong emotional commitment, activate the endogenous motivation of rural teachers' professional development based on emotions, so that they can sprout the seeds of “contributing” on the soil of rural education, and realize the qualitative change from “institutional retention” to “emotional rootedness”.

### 5.3 Emotional resonance—the emotional destiny for rural teachers to strive for excellence

Rural teachers participating in rural education should not only be willing to “remain rooted” and “actively contribute”, but also be able to “strive for excellence”, forming a “breathing with the school, sharing the fate” emotional resonance with the school. The emotional resonance of “breathe together, shared destiny” with the school is the decisive weapon for rural teachers to “excel” in rural education.

Rural teachers to form the same frequency resonance with the school is a continuous process. First of all, rural teachers can actively recognize complex emotions in rural education work. “*I feel that the emotions generated in the process of participating in the construction of school culture are complex and varied and will change with different periods of cultural construction, for example, when the proposed cultural concepts can be accepted and recognized by the majority of teachers, students and parents, I will feel proud and excited; but when I see that the school's development is progressing slowly, it will also generate a sense of powerlessness and frustration.” (B1)* Second, rural teachers complete the transformation of negative to positive emotions based on recognizing complex emotions. “*When I see that everyone is not excited about building school culture, I will try to analyze the reasons for this emotion and follow up with more creative ideas to bring everyone out of the rut.” (D1)* Finally, by always reflecting and regulating their own emotions, rural teachers contribute to the realization of a virtuous coupling and cycle of emotions and work after achieving a balance among various emotions. “*When facing emotional conflicts between myself and others, I will try to stay calm and avoid emotional reactions. When I am able to balance my emotions, I feel more satisfied and confident, and I can focus more on teaching and culture building; of course, the dynamic balance of emotions also enhances my perseverance in the face of challenges and pressures, so that I can be more committed to my educational work, and I can establish a deeper emotional connection with my students and colleagues, and feel the value and meaning of my profession.” (B2)*

Analyzing the theory of Emotional Intelligence, when rural teachers are able to actively use their ability to understand and transform their emotions and their ability to regulate and reflect on their emotions to find their own emotional anchors in the educational space of the rural school, their breathing will keep up with the rhythm of the land, and they will participate in the construction of the school as “insiders”, thus ultimately reaching a dynamic balance of emotions. Thus, a dynamic balance of emotions is finally reached, i.e. the emotional homeostasis of rural education. Once this “emotional stability” is formed, it will not only help to dissolve the emotional alienation brought by the impact of modernity on the rural teachers, but also help to make the rural teachers become “translators of native genes”, so that they can resonate with the school on the basis of the maintenance of native emotional roots, and shape the school-based sense of belonging. This will help rural teachers to become “translators of local genes” and resonate with the school on the basis of maintaining their local emotional roots, shaping their sense of belonging to the school, and thus achieving the ultimate goal of being able to strive for excellence.

## 6 Discussion

### 6.1 Summary of the findings

**Figure 2:**
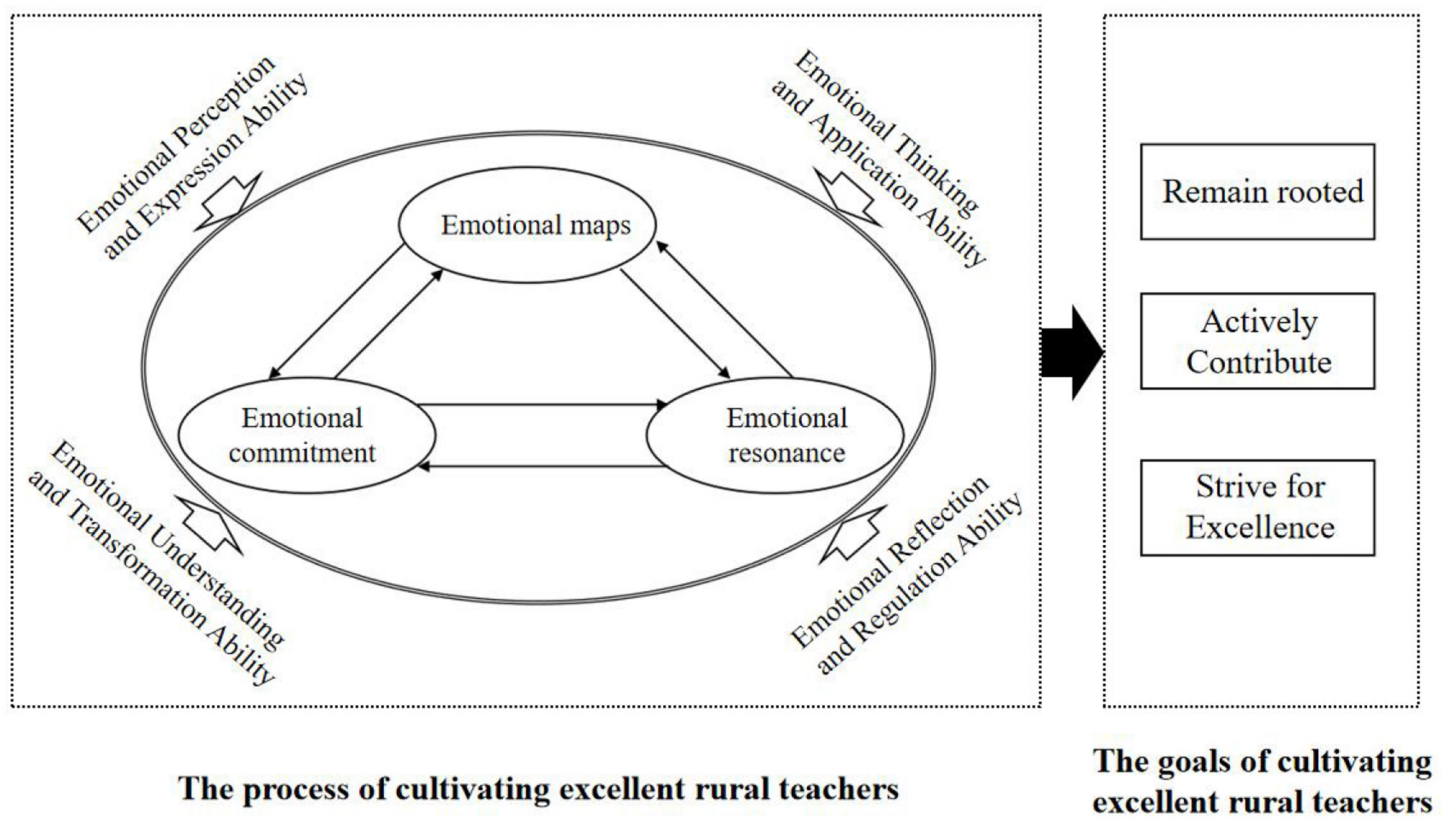
Model of cultivation of excellent rural teacher.

This study, through examining rural teachers' sustained participation in school culture construction, reveals an emotion-driven mechanism underlying the development of excellent teachers characterized by “taking root, dedication, and excellence,” and constructs a two-dimensional emotional intelligence development model with Chinese rural contextual characteristics, as illustrated in Figure 2. The model indicates that, on the horizontal practical dimension, teachers gradually consolidate rural belonging through constructing an emotional map, perceiving collective emotional investment, and forming emotional resonance with the school. On the vertical developmental axis, teachers' emotional intelligence abilities develop progressively across four levels—perception and expression, thinking and application, understanding and transformation, and reflection and regulation—thereby supporting a transformation from emotional participation to emotional taking root and from professional adherence to life integration.

### 6.2 Dialogue with existing literature

#### 6.2.1 Teacher emotion and emotional labor: moving from demand-based regulation to culture-embedded development

Research in organizational and educational psychology has long recognized that work emotions are not incidental, and that emotion regulation constitutes a core requirement in many professions. Emotional labor, conceptualized as the regulation of emotion to meet occupational expectations, provides a foundational lens for understanding how professionals manage affective demands (Grandey, 2000). In the teaching profession, this perspective has been further developed by emotion regulation accounts emphasizing that teachers regulate emotions with specific motives (“why”) and through different strategies (“how”; Taxer and Gross, 2018). The present findings are consistent with this literature insofar as rural teachers continuously engage in emotional work to cope with contextual constraints and interpersonal demands.

At the same time, the present study extends existing accounts by showing that rural teachers' emotional processes are not solely demand-driven regulation in response to stressors; rather, they constitute a culture-embedded developmental trajectory. The “emotional map” identified in this study does not simply reflect momentary coping but functions as a meaning-structured schema through which teachers locate themselves in the rural setting, interpret relationships, and stabilize belonging. This perspective complements emotion regulation theory by highlighting that regulation is intertwined with meaning reconstruction and identity consolidation over time, especially in rural contexts where teachers must repeatedly interpret and re-interpret “why I stay” in relation to local life and school development.

#### 6.2.2 Emotional intelligence: aligning ability models with contextual and developmental evidence in teachers

Emotional intelligence (EI) has been conceptualized as a set of emotion-related abilities concerning the perception, use, understanding, and regulation of emotions (Salovey and Mayer, 1990; Mayer et al., 2000). Parallel approaches have emphasized mixed competency models and workplace performance implications (Goleman, 1995, 2001), as well as trait-like assessment traditions such as the EQ-i (Bar-On, 1997), with evidence linking emotional expression profiles to occupational stress (Bar-On et al., 2000). Measurement work has also supported the validity of EI constructs (Brackett and Mayer, 2003). In teacher populations, perceived EI has been associated with self-efficacy and burnout-related outcomes (Chan, 2004, 2006), and recent studies further suggest that EI and social support shape teachers' intentions to leave the profession (Mérida-López et al., 2022).

The present findings are in line with these studies by indicating that the development of emotional competence supports rural teachers' dedication and long-term engagement. The study contributes additional nuance by specifying a developmental pathway of EI abilities across four progressive levels. Rather than treating EI solely as a relatively stable personal attribute, the vertical axis emphasizes how EI can be cultivated through practice, reflection, and culturally meaningful participation. This process-oriented view is consistent with the broader notion that emotional competence is shaped by both explicit skill development and implicit social–cultural learning (Kosmitzki and John, 1993). Importantly, the rural context appears to provide distinctive developmental affordances: school culture construction repeatedly exposes teachers to emotionally salient events and collective meaning-making, thereby accelerating the transition from basic emotion perception to reflective regulation and meaning transformation.

#### 6.2.3 Retention, organizational support, and commitment: clarifying the “taking root” mechanism

Rural teacher recruitment and retention research indicates that teachers' staying decisions are shaped by a constellation of structural, organizational, and psychosocial factors, including working conditions, induction support, community ties, and professional development opportunities (Hammer et al., 2005; Kersaint et al., 2007; Waterman and He, 2011). Empirical studies across rural contexts further highlight that supportive school environments and social relations contribute substantially to retention (Opoku et al., 2019; Williams et al., 2022), and doctoral research has documented persistent retention challenges in rural high-poverty districts (Boone, 2018; Whaland, 2020). Policy interventions such as bonuses may improve retention, yet their effects can be complex and insufficient for sustainable engagement when relational and cultural conditions remain weak (Candelaria and Urquiola, 2022). Evidence also suggests that burnout and perceived school context are closely tied to teachers' intentions to leave (Li et al., 2021), reinforcing the need for psychological and organizational explanations.

Against this background, the present study clarifies a psychological pathway through which rural teachers convert “staying” into “taking root.” Organizational commitment theory emphasizes affective commitment as a core component of stable attachment to an organization (Meyer and Allen, 1991), and perceived organizational support theory suggests that feeling valued and cared for strengthens reciprocal engagement (Eisenberger et al., 1986), consistent with social exchange theory (Blau, 1986). The “collective emotional investment” component identified in this study can be interpreted as an affective and relational signal that teachers are supported, recognized, and aligned with shared goals, thereby strengthening emotional commitment and sustaining dedication. The concept of emotional resonance extends this literature by highlighting value alignment and narrative integration: teachers' personal life meanings become congruent with the school's cultural development, which can stabilize long-term engagement even under structural constraints.

#### 6.2.4 Resilience and social–ecological resources: situating emotional development in rural beginning teachers

Recent work on Chinese rural beginning teachers emphasizes that resilience development is shaped by social–ecological resources, and that thriving depends on multi-level supports rather than individual coping alone (Sun and Huang, 2024). The present findings converge with this perspective by showing that the emotional pathway toward “taking root” is scaffolded by collective cultural practices and supportive relational environments. The proposed model suggests that participation in culture construction provides both psychological resources and social resources, thereby creating conditions under which teachers can move from survival-oriented adaptation to sustained excellence.

### 6.3 Theoretical contributions

This study contributes to the literature by offering a culture-embedded, psychologically grounded explanation of rural teacher excellence. The findings conceptualize excellence as an emotion-driven developmental process rather than an outcome attributable only to competence accumulation or external incentives. The two-dimensional model integrates a horizontal mechanism with a vertical developmental axis, providing a dynamic framework that links individual emotional abilities with collective cultural practices.

The model also highlights the transformation from emotional participation to emotional taking root and from professional adherence to life integration, thereby enriching commitment and identity-related accounts with a rural-contextual pathway. By connecting EI theory (Salovey and Mayer, 1990; Mayer et al., 2000) with organizational support and commitment frameworks (Eisenberger et al., 1986; Meyer and Allen, 1991; Blau, 1986), the study articulates how emotional competence and organizational–cultural conditions jointly shape long-term dedication and excellence.

### 6.4 Practical implications

The discussion suggests that sustainable cultivation of excellent rural teachers requires interventions that address both psychological capacities and organizational–cultural conditions. Teacher education and in-service professional development may incorporate EI-oriented learning and reflective practices aligned with the four-level developmental pathway identified in this study, especially the capacities for meaning transformation and reflective regulation, which are closely linked to reduced burnout risk and improved professional functioning (Chan, 2006; Mérida-López et al., 2022). Induction and mentoring programs, shown to be beneficial for retention (Waterman and He, 2011), may serve as practical carriers for such EI-oriented development and for building early-career belonging.

At the school level, leadership practices that cultivate perceived support and recognition may strengthen teachers' emotional commitment (Eisenberger et al., 1986) and reinforce reciprocal engagement (Blau, 1986). Culture construction activities that enable genuine participation can make collective emotional investment visible, thereby supporting emotional resonance and long-term “taking root.” At the system level, policy approaches relying primarily on incentives should be complemented by organizational and psychosocial strategies, given that retention is influenced by school context and burnout processes (Li et al., 2021) and that incentive policies may not ensure sustainable effects in isolation (Candelaria and Urquiola, 2022).

### 6.5 Limitations and future research directions

The study is limited by its qualitative design and contextual scope, which may constrain transferability. Future research may adopt comparative designs across different rural regions and school types to examine boundary conditions of the proposed mechanism. Longitudinal studies could capture how emotional maps, collective emotional investment, and emotional resonance develop over time, particularly for beginning teachers whose resilience is shaped by social–ecological supports (Sun and Huang, 2024). Mixed-method approaches may operationalize the core constructs and test their predictive value for burnout, retention intention, and professional performance, given evidence linking perceived context and burnout to quitting intentions (Li et al., 2021). Such validation would strengthen the generalizability and explanatory power of the proposed model.

## 7 Conclusion

This study demonstrates that rural teachers' development into excellent teachers can be understood as an emotion-driven, culture-embedded process. Through participating in school culture construction, teachers construct emotional maps, perceive collective emotional investment, and form emotional resonance with the school, while their emotional intelligence abilities develop progressively across four levels from perception to reflective regulation. This integrated process supports the transformation from emotional participation to emotional taking root and from professional adherence to life integration, thereby underpinning sustained dedication and excellence in rural contexts.

### 7.1 Implications

The conclusions highlight that strengthening rural teacher excellence requires coordinated attention to emotional competence development and supportive school culture. Emotional intelligence theories and evidence in teacher populations (Salovey and Mayer, 1990; Mayer et al., 2000; Chan, 2004, 2006; Mérida-López et al., 2022) indicate that emotion-related abilities and support systems matter for well-being and persistence, while organizational support and commitment frameworks (Eisenberger et al., 1986; Meyer and Allen, 1991; Blau, 1986) clarify how recognition and reciprocal relations consolidate long-term engagement. Rural retention research further suggests that sustainable retention cannot rely solely on material incentives (Hammer et al., 2005; Kersaint et al., 2007; Candelaria and Urquiola, 2022) and should incorporate mentoring and supportive contexts (Waterman and He, 2011; Opoku et al., 2019; Williams et al., 2022). These implications collectively support the view that rural teacher development policies and school practices should foster both emotional competence and collective cultural conditions that enable teachers to truly “take root.”

## Data Availability

The original contributions presented in the study are included in the article/supplementary material, further inquiries can be directed to the corresponding author.
